# Cases of Cerebro-Spinal Affection

**Published:** 1863-08

**Authors:** R. E. McVey

**Affiliations:** Waverly, Ill.


### CASES OF CEREBRO-SPINAL AFFECTION.

By R. E. McVEY, M. D., Waverly, Ill.

I propose to give in outline a brief history of four cases of the so-called Cerebro-Spinal Affection as it has presented itself in my practice of late.

Case I.—Feb. 17th, was called to see Mr. D. S., who had been sick about twelve hours. Taken with a chill, followed by high febrile excitement and pain in the head, back and legs. Upon my arrival I found him in a moribund condition, consequently gave him nothing. Death taking place in about two hours after my arrival.

CASE II.—March 4th, was called to see Mr. C. C. H., who had had a chill. Upon my arrival I found him under high febrile excitement, with pain in the head, back and legs. Suffusion of the eyes, stiffness of the muscles of the neck, and delirium, tongue coated with a thin whitish coat, bowels constipated, pulse about 140 per minute. Treatment, Mercury, Anodynes, Cold Water to the head, Verat. Viride.

7th. Saw him early in the morning; medicine had acted well, pulse 125, skin moist and red, difficulty of passing water, growing weaker. Treatment, Mercury continued, blister to the whole length of the spinal column, cold to the head, Quinine and Dover's every two hours.

8th. Comatose, spasms of the left side of the face. Bled him freely from arm. Continued cold to the head, gave Verat. Viride. Evening, moribund. Death taking place in a short time.

CASE III.—April 29th, called to see Mr. A. H., who had had a chill, followed with high febrile excitement, and redness of the eyes, and pain in the head, back and legs. Not differing particularly from the one given above. Death occurring the fourth day.

CASE IV. June 12th, was called to see Mr. S. F. about noon, who had had a chill early in the morning. When I arrived I found him under high febrile excitement, pain in the head, back and legs, and delirium, pulse 150, eyes congested, tongue slightly coated, bowels constipated, skin hot, with petechiæ over the extremities. Treatment, Mercury, Anodynes every two hours, with cold to the head.

13th. Saw him early in the morning, delirium increased, bowels moved freely twice in the bed, surface cold, pulse had disappeared at the wrist. Treatment, Carb. Ammonia, Stimuli, and blisters to the extremities. Evening, skin warm, pulse about 100. Gave Quinine, grs. vi, Submuriate, grs. iii, every three hours.

14th. Morning, growing weaker. Discontinued the Quinine and Mercury. Gave him Stimuli, Capsicum, and Carb. Ammonia. Evening, still failing. Died at 11, p. m. There were no post mortem examinations.

